# ICTV Virus Taxonomy Profile: *Polycipiviridae*

**DOI:** 10.1099/jgv.0.001241

**Published:** 2019-03-05

**Authors:** Ingrida Olendraite, Katherine Brown, Steven M. Valles, Andrew E. Firth, Yanping Chen, Diego M. A. Guérin, Yoshifumi Hashimoto, Salvador Herrero, Joachim R. de Miranda, Eugene Ryabov, ICTV Report Consortium

**Affiliations:** ^1^​ Department of Pathology, University of Cambridge, Cambridge CB2 1QP, UK; ^2^​ United States Department of Agriculture, Agricultural Research Service, Gainesville, FL 32608, USA; ^3^​ United States Department of Agriculture, Agricultural Research Service, Beltsville, MD 20705, USA; ^4^​ Department of Biochemistry and Molecular Biology, University of the Basque Country (EHU), Biophysics Institute (CSIC-UPV/EHU), Bo Sarriena S/N, 48940 Leioa, Spain; ^5^​ BioMarin Pharmaceutical Inc., San Rafael, CA 94901, USA; ^6^​ Department of Genetics, Universitat de València, Burjassot, Spain; ^7^​ Department of Ecology, Swedish University of Agricultural Sciences, Uppsala 750 07, Sweden

**Keywords:** *Polycipiviridae*, ICTV Report, taxonomy

## Abstract

*Polycipiviridae* is a family of picorna-like viruses with non-segmented, linear, positive-sense RNA genomes of approximately 10–12 kb. Unusually for viruses within the order *Picornavirales*, their genomes are polycistronic, with four (or more) consecutive 5′-proximal open reading frames (ORFs) encoding structural (and possibly other) proteins and a long 3′ ORF encoding the replication polyprotein. Members of species within the family have all been detected in ants or via arthropod transcriptomic datasets. This is a summary of the International Committee on Taxonomy of Viruses (ICTV) Report on the *Polycipiviridae*, which is available at www.ictv.global/report/polycipiviridae.

## Virion

**Fig. 1.**
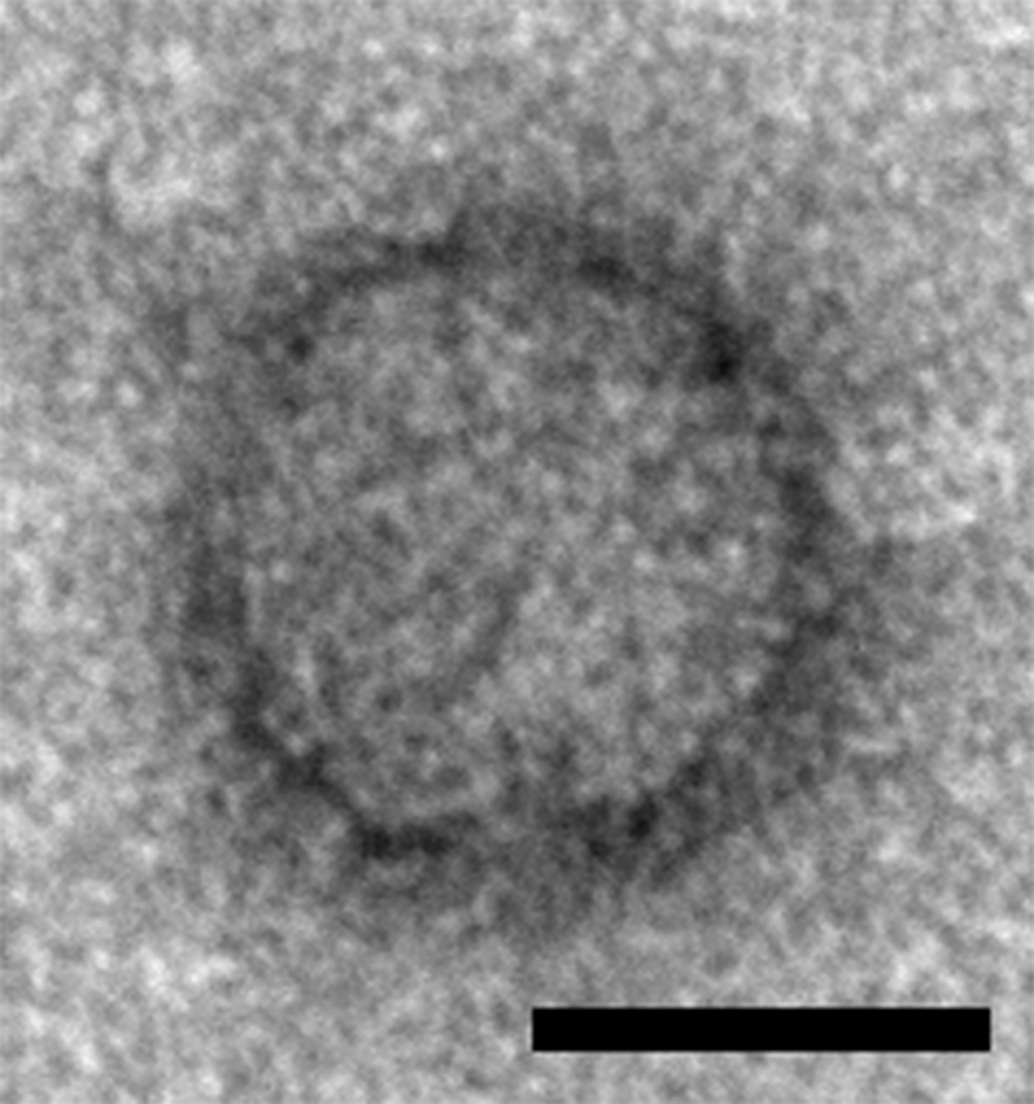
Electron micrograph of a virus-like particle purified from *Solenopsis invicta* worker ants infected with Solenopsis invicta virus 2 (scale bar, 20 nm). Reproduced from [1]; US government material as public domain content.

Icosahedral particles approximately 33 nm in diameter have been observed by electron microscopy in samples prepared from ants infected with Solenopsis invicta virus 2, but not in samples prepared from non-infected ants [1] (Table 1) (Fig. 1). Genomes are picorna-like and appear to encode three jelly-roll fold capsid proteins; thus particles are assumed to be picorna-like, i.e. non-enveloped, with icosahedral pseudo-T=3 symmetry, and comprising 60 copies of each of the ORF1, ORF3 and ORF4 products. However, this has not yet been experimentally confirmed.

**Table 1.** Characteristics of members of the family *Polycipiviridae*

Typical member: Solenopsis invicta virus 2 (MF041813), species *Solenopsis invicta virus 2*, genus *Sopolycivirus*	
Virion	Thought to be non-enveloped, 33 nm in diameter
Genome	10–12 kb of positive-sense, non-segmented RNA
Replication	Not studied; presumed to be similar to other *Picornavirales* members
Translation	Directly from genomic RNA, presumed internal ribosome entry site elements in 5′-untranslated region and intergenic region
Host range	Arthropoda
Taxonomy	Member of the order *Picornavirales*. Includes the genera *Chipolycivirus*, *Hupolycivirus* and *Sopolycivirus*

## Genome

**Fig. 2.**
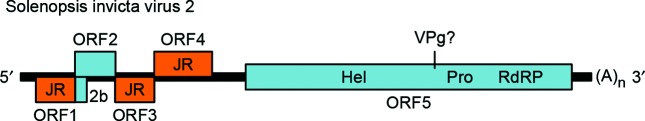
Genome organization of Solenopsis invicta virus 2 (genus *Sopolycivirus)*. The 5′-proximal ORFs encode jelly-roll capsid protein domains (JR; orange), a protein of unknown function (ORF2) and a small predicted transmembrane protein (2b; specific to genus *Sopolycivirus*). The 3′ ORF encodes helicase (Hel), protease (Pro) and RNA-dependent RNA polymerase (RdRP) domains. The genome is polyadenylated and believed to have a viral protein genome-linked (VPg) covalently attached at the 5′ end. Members of the genera *Hupolycivirus* and *Chipolycivirus* have similar genome organizations, except that they lack the 2b ORF.

The positive-sense RNA genome is non-segmented and contains four main 5′-proximal ORFs (ORFs 1–4) and one long 3′ ORF (ORF5) (Fig. 2) [2]. ORF1, ORF3 and ORF4 encode products with homology to picornavirus jelly-roll fold capsid proteins. ORF2 encodes a product of unknown function. ORF5 encodes superfamily III helicase, chymotrypsin-like serine protease and superfamily I RNA-dependent RNA polymerase domains, and is presumed to encode a viral protein genome-linked (VPg) and potentially another protein between the helicase and protease, and one or two additional proteins upstream of the helicase. ORF5 is expected to be proteolytically cleaved by the viral protease, but the cleavage sites have not been mapped. The ORFs are flanked by 5′- and 3′-untranslated regions (UTRs) and a lengthy intergenic region between ORF4 and ORF5. The 5′-UTR and intergenic region are presumed to contain internal ribosome entry site elements to direct translation of ORF1 and ORF5, whereas ORFs 2–4 have been proposed to be expressed via a ribosome reinitiation mechanism. Similar to other members of the order *Picornavirales* [3], the genome has a 3′-poly(A) tail, and is presumed to have a small VPg attached at the 5′-end. Members of the genus *Sopolycivirus* contain an additional ORF (2b) overlapping the 5′-end of ORF2; ORF2b encodes a small protein with a predicted transmembrane domain. Members of at least one species (*Formica exsecta virus 3*) contain a further additional small ORF inserted between ORF2 and ORF3.

## Replication

Replication has not been studied but is assumed to be similar to that of other members of the order *Picornavirales*.

## Pathogenicity

Solenopsis invicta virus 2 appears to establish a chronic, asymptomatic infection in fire ants (*Solenopsis invicta*) and high virus loads have been detected in larvae, pupae, workers and queens [4, 5]. In infected ants, 96 % of Solenopsis invicta virus 2 genome equivalents localize to the midgut section of the alimentary canal, and the virus has been experimentally transmitted to non-infected ants via feeding, suggesting food-borne transmission, probably by trophallaxis [5]. Fire ant infections with Solenopsis invicta virus 2 are associated with negative impacts on queen ants, resulting in significant reductions in fecundity, longer claustral periods and slower growth of newly established colonies [6].

## Taxonomy

The family *Polycipiviridae* includes three genera: *Sopolycivirus*, *Hupolycivirus* and *Chipolycivirus*. Demarcation of genera is based on phylogenetic divergence. All current members were found in ants or via transcriptomic studies of arthropods. *Sopolycivirus* is currently the most species-rich genus, and nearly all members of this genus are associated with ants (family Formicidae).

## Resources

Full ICTV Report on the family *Polycipiviridae*: www.ictv.global/report/polycipiviridae.
